# Complete Chloroplast Genome of the *Eria* Sensu Lato Complex (Orchidaceae): Comparative Analysis and Phylogenetic Relationship

**DOI:** 10.1002/ece3.73342

**Published:** 2026-04-02

**Authors:** Xinyi Wu, Jian Li, Tingzhang Li, Fengxia Tang, Xiaojuan Duan, Wenhui Rao, Meina Wang

**Affiliations:** ^1^ Shenzhen Key Laboratory for Orchid Conservation and Utilization, The National Orchid Conservation Center of China and the Orchid Conservation & Research Center of Shenzhen Shenzhen China; ^2^ Key Laboratory of National Forestry and Grassland Administration for Orchid Conservation and Utilization, The National Orchid Conservation Center of China and the Orchid Conservation & Research Center of Shenzhen Shenzhen China

**Keywords:** chloroplast genome, *Eria* s.l., Orchidaceae, phylogenetic analysis, positive selection, rapid radiation

## Abstract

The *Eria* sensu lato (*Eria* s.l.) complex represents a highly diverse yet taxonomically challenging orchid lineage. To comprehensively elucidate its evolutionary history, we sequenced 14 complete chloroplast genomes and assembled a robust 18‐taxon dataset encompassing its major generic lineages. Comparative genomic analyses revealed that despite overall structural conservation with no rearrangements detected (genome sizes ranging from 150.9 to 159.7 kb), the complex exhibits a lineage‐specific, stepwise degradation of the NAD(P)H dehydrogenase (NDH) complex. This progressive gene loss, coupled with the physical contraction of inverted repeat (IR) boundaries, directly drives plastome miniaturization in specific taxa (e.g., *Eria corneri* and *E. clausa* ). To facilitate fine‐scale species delimitation, we identified seven hypervariable mutational hotspots, which successfully resolved the majority of interspecific relationships with a topology highly congruent to the whole‐plastome tree. Furthermore, selective pressure analysis via branch‐site models detected strong episodic positive selection acting on the *ycf1* gene specifically within the *Pinalia* clade, highlighting potential eco‐physiological adaptations to dynamic epiphytic environments. Finally, phylogenomic reconstructions and divergence dating unveiled a topological incongruence suggesting rapid evolutionary radiation. This concentrated period of early divergence (9.97–5.53 Ma) coincides with Late Miocene paleoclimatic shifts, particularly the intensification of the Asian summer monsoon. Ultimately, this study significantly enriches the plastomic resources for the taxonomically difficult *Eria* s.l. complex, laying a valuable foundation for future evolutionary studies and systematic revisions.

## Graphical abstract

We sequenced and assembled 18 complete chloroplast genomes representing the *Eria* sensu lato complex. Our comparative analyses unveiled stepwise NDH complex degradation, lineage‐specific positive selection on ycf1, and climate‐driven rapid radiation during the Late Miocene. These findings provide robust phylogenomic insights and novel molecular markers for the evolutionary history and conservation of this complex orchid group.

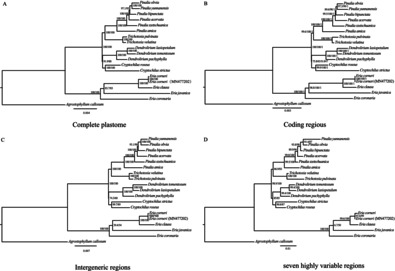

## 1 Introduction

The Orchidaceae subtribe Eriinae encompasses a highly diverse and ecologically significant group of epiphytic and lithophytic orchids, widely distributed across tropical Asia. Historically, its most prominent member, the genus *Eria* Lindl., was treated as a massive and morphologically heterogeneous group, commonly referred to as the *Eria* sensu lato (*Eria* s.l.) complex. Comprising approximately 370 species globally, including 44 species distributed in China (Chen et al. 2009), this group suffered from a broad and poorly defined taxonomic scope (Chase et al. 2003). Extensive morphological variations and widespread convergences have rendered its internal taxonomy notoriously chaotic, leading early researchers to suspect its polyphyletic nature (Seidenfaden 1982; Ng 2002). Subsequent molecular studies utilizing traditional nuclear (*ITS*) and plastid markers (*matK*, *trnL‐F*) conclusively confirmed that *Eria* s.l. is highly polyphyletic. Consequently, it has been segregated into several morphologically distinct, monophyletic genera, including *Pinalia*, *Cryptochilus*, *Dendrolirium*, and *Trichotosia* (Ng et al. 2018). Despite these foundational taxonomic revisions, the deep‐level evolutionary relationships among these generic lineages—as well as the fine‐scale species boundaries within their recently diverged clades—remain ambiguous or weakly supported due to the limited informative sites provided by traditional few‐gene approaches. Consequently, there is a severe lack of comprehensive, genome‐scale phylogenetic studies dedicated to fully resolving the evolutionary history of this complex.

To overcome the limitations of conventional molecular markers, chloroplast (plastid) phylogenomics has emerged as a robust tool for resolving complex plant evolutionary histories. As highlighted in recent perspectives, while the utility of plastomes in resolving deep phylogenetic backbones is well established, much more remains to be explored at the species and population levels (Wang et al. 2024). At these fine‐scale taxonomic levels, whole‐plastome data provide unparalleled resolution for clarifying complex evolutionary relationships in hyper‐diverse orchid groups, such as *Bulbophyllum* and *Cymbidium* (Chen et al. 2024; Simpson et al. 2024). Furthermore, comparative plastomes analysis profoundly unveils lineage‐specific evolutionary dynamics, such as structural rearrangements, the recurrent loss of the *ndh* gene family, and adaptive evolution across varying ecological niches (Xue et al. 2023). Crucially, it also allows for the precise screening of hypervariable mutational hotspots. These tailored loci can be explicitly validated as highly sensitive DNA barcodes to trace maternal ancestry within complex groups (Dong et al. 2025) and resolve closely related species where traditional universal barcodes fail (Liu et al. 2023).

Despite its tremendous taxonomic complexity, the *Eria* s.l. complex remains largely unexplored at the plastome level. To address this critical knowledge gap, we sequenced and assembled the complete chloroplast genomes of 14 key Eriinae species sourced from China. These newly generated sequences were combined with previously published data to assemble a comprehensive 18‐taxon dataset encompassing all major generic lineages within the *Eria* s.l. complex. By leveraging this plastome‐scale data, our study aims to (1) characterize the genomic structure and investigate lineage‐specific gene loss events within *Eria* s.l.; (2) identify and in silico validate novel, hypervariable DNA barcodes tailored for this complex; (3) assess selective pressures to uncover signals of adaptive evolution linked to their epiphytic habitats; and (4) robustly reconstruct the phylogenetic backbone to elucidate the rapid radiation and evolutionary history of the *Eria* s.l. complex. Ultimately, this study provides a foundational phylogenomic framework for the future conservation, systematic revision, and evolutionary study of Eriinae.

## 2 Materials and Methods

### 2.1 Plant Materials and DNA Extraction

The plant samples used in this study were sourced from the National Orchid Conservation & Research Center of Shenzhen, with corresponding specimens stored in the herbarium of the same institution. Details of the samples, including 14 species newly sequenced for this research and 5 species obtained from the National Center for Biotechnology Information (NCBI), are provided in Table 1 (Table S1). Genomic DNA was extracted from leaf tissues using the Cetyltrimethylammonium bromide (CTAB) protocol (Doyle and Doyle 1987). To generate high‐quality sequence data, paired‐end sequencing (150 bp read length) was performed on an Illumina Novaseq X platform (Illumina, San Diego, CA, USA) at Novogene (Beijing, China), yielding approximately 8 Gb of raw data per species.

**TABLE 1:** Complete chloroplast genome features of different *Eria* s.l. species.

Species	Cp genome	LSC	IR	SSC	GC content (%)				Number of	Protein‐coding	tRNA	rRNA
	Length (bp)	Length (bp)	Length (bp)	Length (bp)	Total	LSC	IR	SSC	Genes	Genes	Genes	Genes
*Cryptochilus roseus*	159,479	87,113	26,917	18,532	37.0	34.7	43.1	30.0	131	85	38	8
*Cryptochilus strictus*	158,615	87,735	26,207	18,466	36.9	34.6	43.2	30.1	131	85	38	8
*Dendrolirium lasiopetalum*	158,740	88,062	26,213	18,252	37.0	34.7	43.2	30.3	131	85	38	8
*Dendrolirium pachyphylla*	159,715	87,232	26,983	18,517	37.1	34.8	43.1	30.1	131	85	38	8
*Dendrolirium tomentosum*	159,112	87,730	26,644	18,094	37.1	34.8	43.1	30.3	131	85	38	8
*Eria corneri* (MN477202)	150,956	85,316	25,437	16,323	37.3	34.9	43.5	30.0	127	81	38	8
*Eria corneri*	150,973	85,979	25,502	13,973	37.3	34.9	43.5	30.1	127	81	38	8
*Eria coronaria*	156,129	85,978	25,510	13,975	37.4	34.9	43.5	30.1	131	85	38	8
*Eria javanica*	156,807	84,475	26,942	17,770	37.3	35.0	43.1	30.5	131	85	38	8
*Eria clausa*	152,513	85,995	26,926	16,960	37.3	35.0	43.1	30.4	130	84	38	8
*Pinalia acervata*	159,286	87,211	26,808	18,459	37.0	34.7	43.0	30.1	131	85	38	8
*Pinalia amica*	158,566	86,807	26,655	18,449	37.1	34.8	43.1	30.1	131	85	38	8
*Pinalia bipunctata*	159,161	87,098	26,786	18,491	37.0	34.8	43.1	30.1	131	85	38	8
*Pinalia obvia*	159,466	87,333	26,826	18,481	36.9	34.7	43.1	30.1	131	85	38	8
*Pinalia szetschuanica*	158,737	87,150	26,540	18,507	37.0	34.7	43.2	30.1	131	85	38	8
*Pinalia yunnanensis*	158,486	86,486	26,759	18,482	37.0	34.7	43.1	30.1	131	85	38	8
*Trichotosia pulvinata*	159,201	87,246	26,929	18,097	37.1	34.8	43.1	30.5	131	85	38	8
*Trichotosia velutina*	159,105	87,346	26,796	18,167	37.1	34.8	43.1	30.4	131	85	38	8

### 2.2 Chloroplast Genome Assembly and Annotation

Chloroplast genomes were assembled using GetOrganelle v1.7.5 (Jin et al. 2020) with the default parameters ‐R 15 ‐k 21,45,65,85,105 and the ‐F embplant_pt flag, utilizing *Trichotosia velutina* (OR544616) and *Eria corneri* (MN477202) as reference. Initial genome annotation was performed using the Plastid Genome Annotator (PGA) (Qu et al. 2019). To ensure the accuracy of start/stop codons and exon‐intron boundaries, the annotation results were manually inspected and corrected by comparing them with homologous genes from the reference species. Furthermore, tRNA genes were further verified using tRNAscan‐SE v2.0 (Lowe and Chan 2016) with default settings.

### 2.3 Comparative Analysis of Cp Genomes

The online tool CPJSdraw was uesd to examine and visualize the expansion and contraction of the LSC, SSC, IRa, and IRb boundaries in the chloroplast genomes of *Eria* s.l. species (Li, Guo, et al. 2023). To assess whole‐plastome sequence divergence and detect potential structural rearrangements (e.g., gene inversions or translocations), multiple sequence alignment of the chloroplast genomes of the 17 species was conducted using the mVISTA program (https://genome.lbl.gov/vista/mvista, accessed on 12 January 2026) in the Shuffle‐LAGAN mode, with the *Pinalia obvia* sequence serving as the reference (Brudno et al. 2003).

### 2.4 Repeat Sequence Analysis

Long repetitive sequences (LSRs), including forward, reverse, complement, and palindromic repeats, were detected using the REPuter program (https://bibiserv.cebitec.uni‐bielefeld.de/reputer, accessed on 10 February 2026), with a minimum repeat length of 30 bp and a Hamming distance of 3 (Kurtz et al. 2001). Simple sequence repeats (SSRs) were identified with the MISA‐web Perl script (Beier et al. 2017), applying minimum repeat unit thresholds of 10, 5, 4, 3, 3, and 3 for mono‐, di‐, tri‐, tetra‐, penta‐, and hexa‐motif microsatellites, respectively.

### 2.5 Nucleotide Diversity

To evaluate sequence divergence and identify mutational hotspots across the *Eria* s.l. plastomes, nucleotide diversity (Pi) analysis was conducted. The shared protein‐coding genes (CDS) and intergenic spacer (IGS) regions were extracted using the “Pi” subcommand in CPStools (Huang et al. 2024). Prior to the calculation of Pi values, sequences for each extracted region were independently aligned using MAFFT v7.505 (Katoh and Standley 2013). Following the alignment and calculation, regions exhibiting exceptionally high nucleotide diversity (Pi > 0.048) were selected as potential candidate DNA barcodes. The discriminatory power of these combined hypervariable loci was subsequently evaluated through phylogenetic reconstruction.

### 2.6 Gene Selective Pressure Analysis

To assess selective pressures on plastid CDS, we calculated the synonymous (Ks), non‐synonymous (Ka) substitution rates, and their ratio (Ka/Ks). First, 75 shared CDS were extracted from the annotated GenBank files using the python script get_annotated_regions_from_gb.py (available at https://github.com/Kinggerm/PersonalUtilities/). The sequences were aligned based on amino acid translations using MAFFT v7.505 and subsequently back‐translated to nucleotides with internal stop codons removed via Biopython (Cock et al. 2009). Pairwise Ka and Ks values were calculated using KaKs_Calculator v3.0 (Zhang 2022) under default settings, designating *Pinalia amica* as the reference species. Genes with undefined Ka/Ks ratios (Ks = 0) were excluded, retaining 42 genes. A Ka/Ks ratio > 1 indicates positive (adaptive) selection, while < 1 indicates purifying selection.

Furthermore, the branch‐site model in the codeml program of PAML v4.10.7 (Yang 2007) was used to detect episodic positive selection driving lineage divergence. Based on the reconstructed phylogeny, the five major generic lineages within *Eria* s.l. were successively designated as foreground branches. Likelihood Ratio Tests (LRTs) were performed comparing the alternative model (Model 2, NSsites = 2, fix_omega = 0) to the null model (Model 2, NSsites = 2, fix_omega = 1, omega = 1) (Zhang et al. 2005). Statistical significance was assessed using a Chi‐square distribution (d*f* = 1).

### 2.7 Phylogenetic Analysis

To resolve the relationships among the 18 *Eria* s.l. species, four datasets were constructed: (1) complete chloroplast genomes; (2) 79 shared CDS; (3) 133 IGS regions; and (4) concatenated hypervariable regions. Sequences extracted via PhyloSuite (Zhang et al. 2020) were aligned using MAFFT v7.505 and trimmed with trimAl v1.2 (Capella‐Gutiérrez et al. 2009). Maximum Likelihood (ML) phylogenies for all four datasets were reconstructed using IQ‐TREE v1.6 (Nguyen et al. 2015). The optimal substitution model, TVM + F + R2, was automatically identified by ModelFinder according to the BIC (Kalyaanamoorthy et al. 2017). Node support was comprehensively assessed using a combination of the SH‐aLRT test (1000 replicates) and standard non‐parametric bootstrapping (1000 replicates). To corroborate the ML topology, Bayesian Inference (BI) was performed exclusively on the 79 CDS dataset using MrBayes v3.2 (Ronquist et al. 2012). Two independent MCMC runs of 50,000,000 generations were executed, sampling every 1000 generations until convergence, with a 25% burn‐in.

### 2.8 Divergence Time Estimation

To estimate species divergence times, an expanded 64‐taxon plastome dataset (Table S5) was analyzed. From this dataset, 67 shared CDS were extracted, aligned using MAFFT v7.505, and concatenated. A time‐calibrated BI phylogeny was first reconstructed using MrBayes v3.2 based on models determined by ModelTest‐NG (Darriba et al. 2020), utilizing two MCMC runs of 1000,000 generations and a 25% burn‐in. Based on this topology, molecular dating was performed using the Bayesian relaxed clock method in the mcmctree program (PAML v4.10.7). Two reliable orchid fossils constrained the crown clades of *Dendrobium* (23.2 Ma; Conran et al. 2009) and *Goodyerinae* (15.0 Ma). Secondary calibration priors were applied to the stem node of Orchidaceae/monocots (112.0 Ma; Ramírez et al. 2007) and the most recent common ancestor of all extant orchids (90.0 Ma; Givnish et al. 2015, 2016; Xiang et al. 2016).

## 3 Results

### 3.1 Chloroplast Genome Features

The chloroplast genomes of the 18 *Eria* s.l. species analyzed in this study exhibited a typical double‐stranded, circular, quadripartite structure. The genome sizes varied significantly, ranging from 150,956 bp in *Eria corneri* (MN477202) to 159,715 bp in *Dendrolirium pachyphylla*. The overall GC content of these genomes ranged from 36.9% to 37.4%. Most species contained 131 genes, including 85 CDS, 8 rRNA genes, and 38 tRNA genes. However, two species exhibited a reduced gene complement due to specific gene losses: *Eria clausa* contained 130 genes (with 84 CDS), while *E. corneri* displayed a more reduced plastome comprising only 127 genes (with 81 CDS). The lengths of the large single‐copy (LSC) and small single‐copy (SSC) regions showed interspecific variation, ranging from 84,475 to 88,062 bp and 13,973 to 18,532 bp, respectively. The IR regions were relatively conserved, with lengths ranging from 25,437 to 26,983 bp. The GC content within each region was as follows: 43.0%–43.5% in the IR regions, 30.0%–30.4% in the SSC region, and 34.6%–35.0% in the LSC region (Table 1).

**FIGURE 1:**
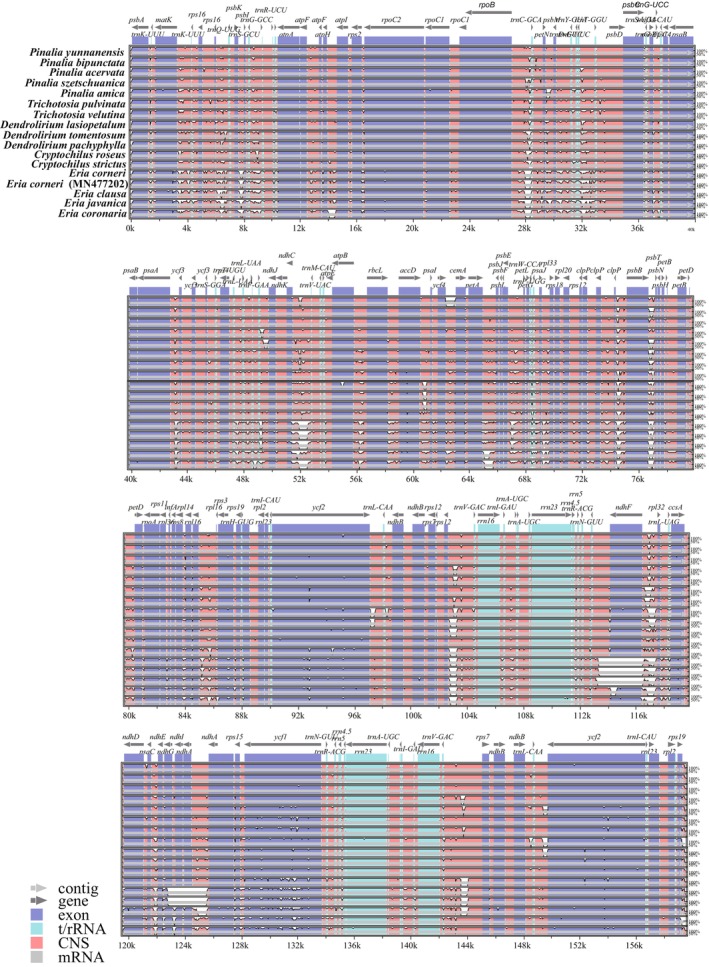
Comparison of 18 *Eria* s.l. chloroplast genomes using the mVISTA program, with the annotation of *Pinalia obvia* serving as the reference.

In the 18 chloroplast genomes analyzed, all genes appeared as single copies in the LSC or SSC regions. However, 17 gene duplications were observed in the IR regions, including six tRNA genes and nine CDS. Across the entire plastome, three genes (*ycf3*, *clpP*, and *rps12*) each contained two introns, while the others possessed a single intron. The majority of variable sites were found in intergenic spacers, suggesting a higher evolutionary rate in these non‐coding regions compared to coding regions. Additionally, the IR regions exhibited a higher degree of conservation than the LSC and SSC regions across all species. These results suggest that the chloroplast genome structure and gene sequences are highly similar across these species, with no gene rearrangements detected. Notably, however, *E. corneri* and *E. clausa* showed significant sequence divergence and specific gene deletions in the SSC region corresponding to the NDH complex. Specifically, *E. clausa* exhibited an isolated deletion of the *ndhF* gene, whereas *E. corneri* displayed a more extensive loss, lacking the *ndhA*, *ndhF*, *ndhG*, and *ndhI* genes (Figure 1).

### 3.2 Contraction and Expansion of Inverted Repeats

**FIGURE 2:**
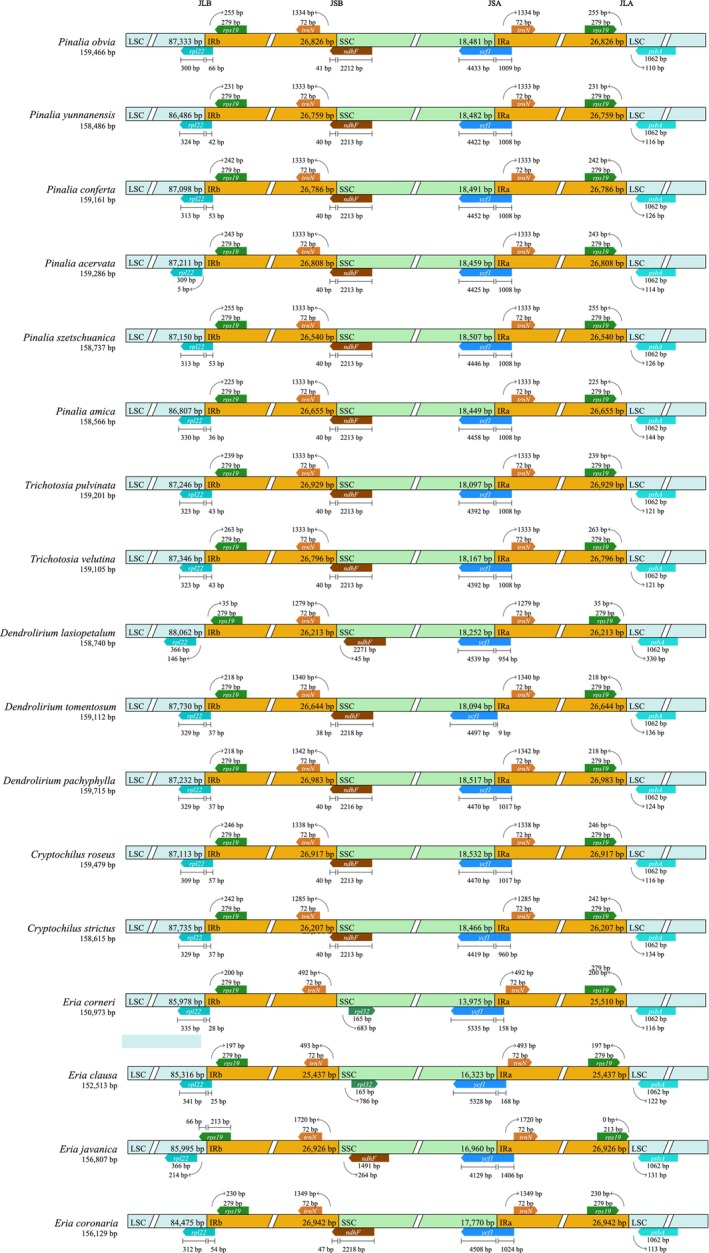
Comparison of the boundaries between the LSC, SSC, and IR regions among 17 *Eria* s.l. chloroplast genomes.

In this study, the contractions and expansions of the IR regions were systematically analyzed by comparing the four distinct junctions (JLB, JSB, JSA, and JLA) across 17 orchid chloroplast genomes (Figure 2). Although the overall genomic structures are highly conserved, significant variations were observed in the distribution and distances of genes adjacent to these junctions. Two distinct patterns were identified at the LSC/IRb (JLB) boundary. In 14 of the analyzed species, the *rpl22* gene spans across the junction. Conversely, in *Pinalia* acervata, *Dendrolirium lasiopetalum*, and *Eria javanica*, this gene is located entirely within the LSC region, positioned at a distance ranging from 5 to 214 bp away from the IRb boundary. The IRb/SSC (JSB) junction was relatively stable among these orchids; in the majority of the genomes, the *ndhF* gene spanned this boundary. A notable exception occurred in *E. Corneri* and *E. clausa* , where *ndhF* was completely absent at the junction. Furthermore, the SSC/IRa (JSA) and IRa/LSC (JLA) junctions exhibited high conservation across all evaluated genomes. The *psbA* gene was fully contained within the LSC region in all species, positioned at 110–330 bp from the IRa/LSC (JLA) boundary.

### 3.3 Repeat Sequence Analysis

**FIGURE 3:**
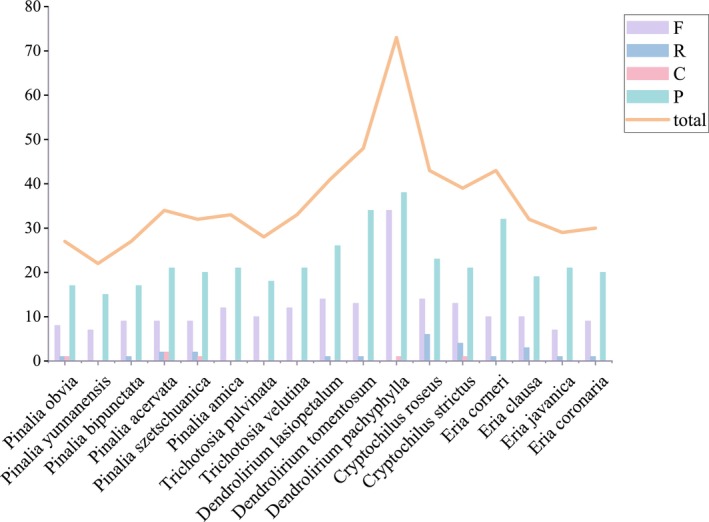
Frequency of four types of long repeats, Forward (F), Reverse (R), Complement (C), and Palindromic (P), in the chloroplast genomes of 17 *Eria* s.l. species.

A total of 614 LSRs were detected across the 17 *Eria* s.l. plastomes, comprising forward (F), reverse (R), complement (C), and palindromic (P) repeats (Figure 3). Palindromic (62.54%) and forward (32.57%) repeats were the most abundant, while reverse and complement repeats were rare. *Dendrolirium pachyphylla* contained the highest number of LSRs (73), whereas *Pinalia yunnanensis* possessed the fewest (22). Only four species (*Cryptochilus strictus*, *Pinalia acervata*, *Pinalia obvia*, and *Pinalia szetschuanica*) exhibited all four repeat types. Conversely, *Pinalia amica*, *P. yunnanensis* , *Trichotosia pulvinata*, and *T. velutina* completely lacked both reverse and complement repeats.

**FIGURE 4:**
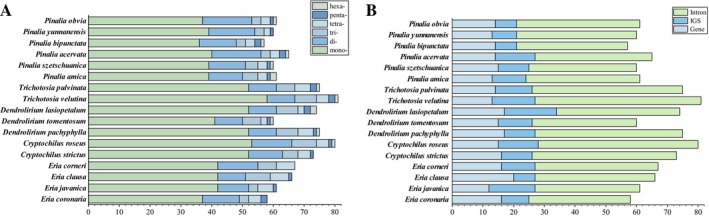
Simple sequence repeats (SSRs) in the chloroplast genomes of 17 *Eria* s.l. species. (A) Number of different SSR motifs (mono‐, di‐, tri‐, tetra‐, penta‐, and hexa‐). (B) Frequency of SSRs in the intergenic regions (IGS), protein‐coding genes, and introns.

A total of 1134 SSRs were identified, ranging from 57 in *Pinalia bipunctata* to 81 in *T. velutina* . Mononucleotide repeats were the most prevalent (66.4%), followed by dinucleotides (17.2%), with longer motifs (tri‐ to hexanucleotides) being progressively less common (Figure 4). Consistent with typical plastome compositions, these SSRs were overwhelmingly A/T‐rich. Regarding their genomic distribution, the majority of SSRs (61.29%) were concentrated in the IGS regions, compared to coding (22.40%) and intronic (16.31%) regions. Notably, our analysis revealed a unique lineage‐specific feature addressing the diversity of SSRs: *E. corneri* was the only species completely lacking both pentanucleotide and hexanucleotide repeats, a structural reduction that coincides with its miniaturized genome size.

### 3.4 Nucleotide Diversity and DNA Barcoding Validation

**FIGURE 5:**
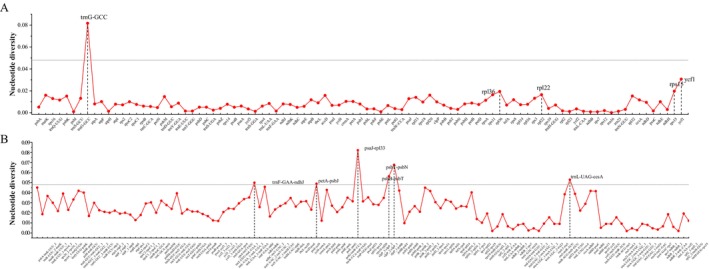
Nucleotide diversity (Pi) across the complete chloroplast genomes of 17 *Eria* s.l. species. (A) Comparison of the nucleotide variability (PI) among CDS regions. (B) Comparison of the nucleotide variability among IGS regions.

To examine mutation hotspots within the 17 *Eria* s.l. chloroplast genomes, Pi values were calculated using CPStools for both shared single‐copy genes and intergenic regions (Figure 5, Table S2). The analysis showed higher divergence in the single‐copy regions (LSC and SSC) and greater conservation in the IR region. Furthermore, the Pi values was generally higher in intergenic regions than in coding regions. Among the genes studied, *trnG‐GCC* (0.08161), *ycf1* (0.03068), *rps15* (0.01975), *rpl36* (0.01938), and *rpl22* (0.01652) exhibited the highest Pi values. Based on a threshold of Pi > 0.048, a total of seven hypervariable regions were identified: one gene (*trnG‐GCC*, 0.08161) and six IGS regions (*psaJ‐rpl33*, 0.08222; *psbT‐psbN*, 0.06772; *psbB‐psbT*, 0.05634; *trnL‐UAG‐ccsA*, 0.05294; *trnF‐GAA‐ndhJ*, 0.04992; and *petA‐psbJ*, 0.04895).

Subsequently, to rigorously assess their utility for species resolution, a phylogenetic tree was reconstructed utilizing the concatenated sequences of these seven specific high‐Pi regions. Remarkably, the resulting topology was identical to those derived from the three comprehensive datasets (the complete chloroplast genomes, the 79 plastid CDS, and the 133 IGS regions), yielding robust branch support across the vast majority of internal nodes (Figure 7).

### 3.5 Positive Selection Analysis

**FIGURE 6:**
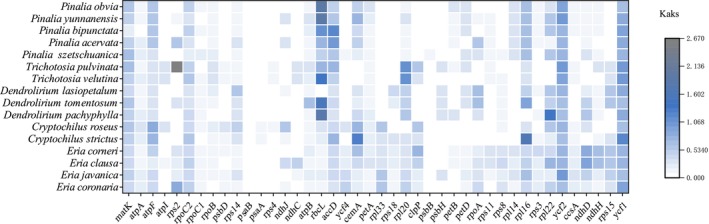
Ka/Ks ratio of 42 CDS of 17 *Eria* s.l. chloroplast genomes. The ratios were calculated using Pinalia amica as the reference species. The x‐axis displays the 42 analyzed CDS (genes with undefined Ka/Ks ratios due to Ks = 0 were excluded), and the y‐axis represents the 16 compared species.

Comparative analysis of selective pressures among 17 *Eria* s.l. species revealed differential patterns of evolutionary constraint on plastid CDS. The ratio of non‐synonymous (Ka) to synonymous (Ks) substitution rates was calculated for 75 shared CDS across the analyzed species, using *P. amica* as the reference. The Ka/Ks ratios for most genes were substantially less than 1, with an overall average of 0.2520 (Figure 6, Table S3). However, specific genes exhibited notably elevated Ka/Ks values compared to the background average. Among all analyzed genes, *ycf1* displayed the highest sequence divergence, with its maximum pairwise Ka/Ks ratio slightly exceeding 1 (1.0011). Other genes exhibiting relatively high, yet purifying, Ka/Ks values included *accD* (0.7588), *clpP* (0.6974), and *matK* (0.5055).

To test whether adaptive evolution drove the divergence of key lineages, we used the branch‐site model in codeml to detect signals of positive selection. Based on the reconstructed phylogeny, the five genera investigated in this study were successively designated as foreground branches. Following LRTs on 75 CDS, the *ycf1* gene was identified as having undergone episodic positive selection specifically on the *Pinalia* branch. A comparison of the alternative and null models revealed a significant increase in the log‐likelihood value for *ycf1* (ln *L*_1_ = −10583.60, ln *L*_0_ = −10592.84), with an LRT statistic (2Δln*L*) of 18.48 (df = 1, *p* < 0.001) (Table S4).

### 3.6 Phylogenetic Analysis and Divergence Times

**FIGURE 7:**
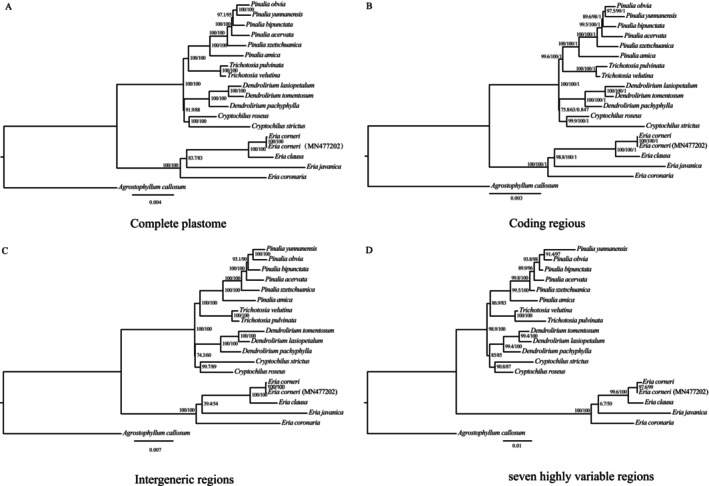
Phylogenetic trees of *Eria* s.l. species based on four datasets: (A) complete chloroplast genomes, (B) 79 shared protein‐coding sequences (CDS), (C) 133 intergenic spacer (IGS) regions, and (D) 7 highly variable region concatenation. *Agrostophyllum callosum* as the outgroup. Numbers at the nodes represent SH‐aLRT/ML bootstrap support values. For tree B, the third number represents the Bayesian posterior probability (PP).

Phylogenetic analyses utilizing ML across four distinct datasets (A: complete chloroplast genomes; B: 79 shared CDS; C: 133 IGS regions; and D: concatenated seven highly variable regions), complemented by BI on the CDS dataset (B), yielded highly congruent topologies (Figure 7). The sampled *Eria* s.l. taxa formed a strongly supported monophyletic clade and were classified into five major generic clades. Within this complex, the genus *Eria* s.s. is positioned at the base of the tree, forming a sister group to the clade containing the other four genera. In these unconstrained phylogenies, *Pinalia* and *Trichotosia* form one clade (e.g., 100/100 support in Tree A), while *Cryptochilus* and *Dendrolirium* form another (e.g., 91.9/88 support in Tree A), with these two clades being sister groups.

**FIGURE 8:**
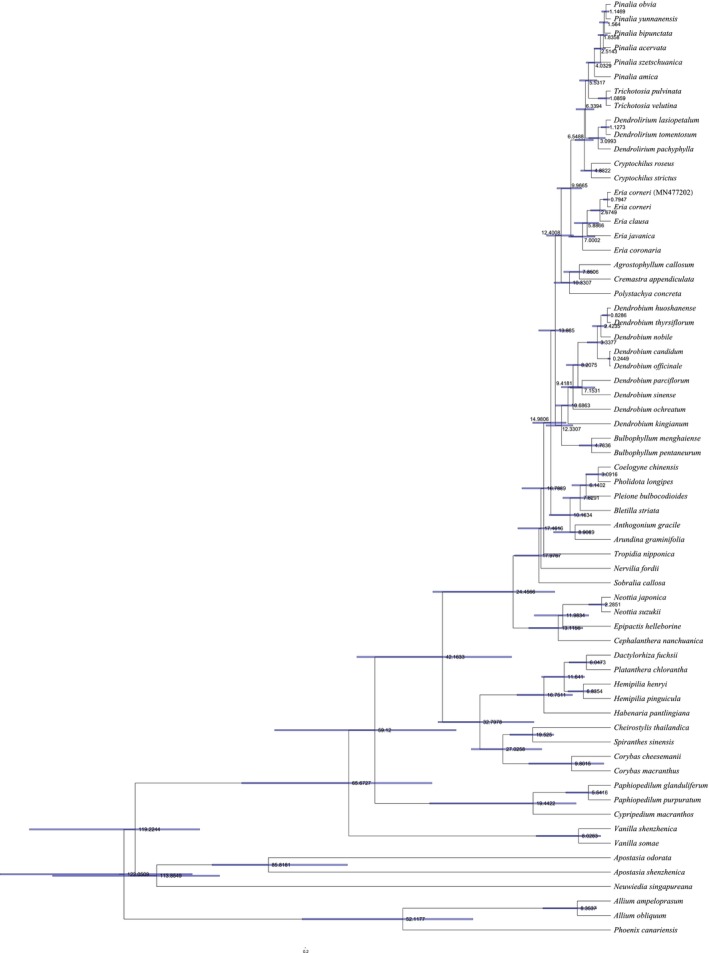
Divergence time estimation of *Eria* s.l. and related taxa. The time‐calibrated phylogeny was inferred based on 67 CDS from 64 taxa. Numbers at the nodes indicate the estimated divergence times in millions of years ago (Ma).

Conversely, the time‐calibrated chronogram (Figure 8) inferred a slightly different, grade‐like topology for the generic divergence following the initial split of *Eria*. Divergence time estimation indicated that the stem age of the *Eria* s.l. clade was approximately 12.40 million years ago (Ma). The initial crown diversification commenced in the Late Miocene, with the genus *Eria* diverging from the ancestor of the remaining four genera at 9.97 Ma. Within the remaining clade, *Cryptochilus* diverged next at approximately 6.55 Ma. This was followed by the divergence of *Dendrolirium* at 6.34 Ma. The terminal split between the sister genera *Pinalia* and *Trichotosia* occurred near the Miocene–Pliocene boundary at 5.53 Ma. Finally, within the *Pinalia* lineage, crown diversification initiated around 4.03 Ma, with *P. amica* occupying the most basal position, while terminal species such as *P. bipunctata* , *P. yunnanensis* , and *P. obvia* diverged much more recently during the Pleistocene (c. 2.51–1.15 Ma).

## 4 Discussion

### 4.1 Chloroplast Genome Characteristics and NDH Loss

In this study, we report the complete chloroplast genomes of 14 *Eria* s.l. species for the first time and compare them with those of four previously published species. The chloroplast genomes of the 18 *Eria* s.l. species exhibited a highly conserved quadripartite structure, gene order, and overall GC content. The absence of major structural rearrangements or inversions, as evidenced by our comparative analyses, indicates a stable evolutionary history of the plastome macrostructure within this complex, which is consistent with patterns observed in most epidendroid orchids.

In higher plants, the chloroplast NAD(P)H dehydrogenase (NDH) complex mediates cyclic electron flow around photosystem I, which is essential for alleviating photo‐oxidative stress and maintaining photosynthetic efficiency (Shikanai 2016). A fully functional NDH complex comprises 11 plastid‐encoded subunits (*ndhA–K*). Our comparative genomic analysis revealed a fascinating intrageneric divergence in the retention of these *ndh* genes within the *Eria* genus. Most of the examined species, including *E. coronaria* and *E. javanica* , retain a complete and highly conserved set of all 11 plastid *ndh* genes. This indicates that the complete NDH pathway was functionally conserved in the common ancestor of the *Eria* complex. However, a remarkable pattern of lineage‐specific genomic reduction was observed within a distinct and well‐supported clade comprising *E. corneri* and *E. clausa* . While *E. clausa* exhibited only the isolated loss of the *ndhF* gene, its sister species, *E. corneri*, displayed much more severe degradation, lacking the *ndhA*, *ndhF*, *ndhG*, and *ndhI* genes. This substantial divergence within a single subclade provides compelling evidence for a stepwise degradation model of the NDH complex. Our findings align perfectly with the evolutionary trajectory recently revealed in *Cymbidium*, where the recurrent loss of *ndhF* at dynamic IR boundaries triggered progressive degradation and widespread pseudogenization of the entire NDH complex across independent lineages (Chen et al. 2025; Kim et al. 2015). We hypothesize that the initial structural disruption of *ndhF* occurred in the common ancestor of *E. corneri* and *E. clausa* .

Similar patterns of *ndh* gene loss have been widely observed in other genera within the Epidendroideae subfamily, such as *Calanthe*, *Dendrobium*, and *Epidendrum* (Chen et al. 2020; Xue et al. 2023; Zhao et al. 2023), implying that the NDH complex is not strictly essential for specific photosynthetic orchids. Sanderson et al. (2015) proposed that the inactivation of the *ndh* gene family might be strongly associated with an epiphytic lifestyle. To optimize photosynthesis under fluctuating canopy light environments, these epiphytes might rely on alternative physiological mechanisms, such as the PGR5/PGRL1‐dependent CEF pathway, to compensate for the loss of NDH function. Additionally, while some orchids have shown a transfer of *ndh* genes from the chloroplast to the mitochondrial (mt) or nuclear genome to maintain functionality, there is currently no definitive evidence directly linking this intracellular gene transfer to the initial loss of these genes in orchid chloroplast genomes (Lin et al. 2015). The rapid and progressive dismantling of the NDH complex in *E. corneri* and *E. clausa* thus highlights a dynamic genomic adaptation to their specific epiphytic niches.

### 4.2 Dynamics of IR Boundaries

Despite the overall structural stasis within the *Eria* s.l. complex, significant variations in genome size (ranging from 150,956 bp to 159,715 bp) were observed. Previous studies have shown that the contraction and expansion of IR boundaries are common events in plastid evolution, significantly influencing variations in plastid length and gene content across angiosperms (Raubeson et al. 2007). The size of the orchid plastome is also strongly related to changes in IR boundaries (Guo et al. 2021). Numerous studies have pointed out that instability at the IR/SSC junctions in orchids is strongly correlated with the loss of the *ndhF* gene (Kim et al. 2015; Niu et al. 2017).

In our study, only the LSC/IRb and IRb/SSC junctions exhibited two types among the 18 species across the four boundaries, while the other two (SSC/IRa and IRa/LSC) remained more conserved and stable (Figure 2). Notably, the variation in the JSB in *E. corneri* and *E. clausa* may be potentially linked to the absence of the *ndhF* gene, which likely contributes to the observed reduction in their chloroplast genome size compared to other *Eria* s.l. species. The connection between the *ndh* gene loss and genome size reduction has also been reported in the genera *Chiloschista*, *Bulbophyllum*, and *Renanthera* (Liu et al. 2023; Tao et al. 2023; Wu et al. 2024).

While the position of the boundaries, especially the expansion and contraction of the IR region, may provide insights into lineage evolution, our observations do not offer sufficient information to clarify the evolutionary relationships among taxa. Consequently, resolving the complex taxonomy of this group necessitates further sampling and more comprehensive comparisons relying on highly variable regions or whole‐plastome data.

### 4.3 Repeat Sequence Analysis

Repeat sequences, including SSRs and LSRs, are fundamental drivers of chloroplast genome rearrangement, sequence divergence, and mutation generation (Cavalier‐Smith 2002). In the examined *Eria* s.l. plastomes, palindromic and forward repeats were the most abundant LSRs (Figure 3). These large repeats are frequently associated with mutational hotspots and promote structural diversity in intergenic regions across orchid plastomes.

Regarding SSRs, the motifs identified in our study were highly A/T‐biased, consistent with observations in other plant plastomes (Jiang et al. 2022; Li, Wang, et al. 2023). While this compositional bias is a ubiquitous phenomenon attributed to the overall low GC content of plastomes, the genomic distribution of these repeats provides significant evolutionary insights. As Ellegren (2004) demonstrated, the distribution and variation of microsatellites within genomes directly reflect underlying selective pressures. In our analysis, we observed a highly non‐random distribution, with the vast majority of SSRs (61.29%) concentrated in the IGS regions, while their frequency was heavily suppressed in CDS (Figure 4). This distribution pattern strongly implies that stringent purifying selection acts to eliminate repeat expansions or contractions within coding regions to maintain reading frame integrity. Conversely, non‐coding regions experience relaxed purifying selection, permitting the accumulation of these mutational elements to drive sequence divergence (Liu et al. 2025).

A particularly noteworthy finding in our analysis is the absence of complex SSRs (pentanucleotide and hexanucleotide repeats) in *E. corneri*. As discussed previously, *E. corneri* possesses the smallest chloroplast genome among the sampled taxa, primarily due to the extensive degradation of the NDH complex and the contraction of its IR regions. The concurrent lack of complex, long‐motif SSRs in this species suggests a potential correlation between overall plastome miniaturization and a general suppression or loss of complex repetitive elements. A similar coupled reduction of genome size and repeat complexity has been documented in other highly specialized or reduced plastomes (Wicke et al. 2011). Ultimately, the abundant and variable repeat sequences identified in these *Eria* s.l. species represent an invaluable genomic resource, providing excellent candidate loci for developing highly polymorphic molecular markers for future population genetics and phylogeographic studies.

### 4.4 Phylogenetic Validation of Specific DNA Barcodes

Traditional universal DNA barcodes often fail to resolve the complex taxonomy of closely related Orchidaceae species, necessitating the identification of supplementary or lineage‐specific markers (Li et al. 2021). Through Pi analysis of the *Eria* s.l. plastomes, we identified seven hyper variable mutational hotspots (Pi > 0.048): one tRNA gene (*trnG‐GCC*) and six IGS (*psaJ‐rpl33*, *psbT‐psbN*, *psbB‐psbT*, *trnL‐UAG‐ccsA*, *trnF‐GAA‐ndhJ*, and *petA‐psbJ*). Notably, while the *ycf1* gene is frequently utilized as a universal barcode in many plant lineages, it exhibited lower variability (Pi = 0.03068) than our identified top‐tier hotspots (Figure 5). The identification of these seven superior loci demonstrates the critical value of screening whole plastomes to discover lineage‐specific markers, particularly since standard universal barcodes frequently fail to provide adequate resolution in complex orchid groups (Li et al. 2021; Simpson et al. 2024).

By reconstructing a phylogenetic tree utilizing the concatenated alignment of these seven novel hotspots, we found that the resulting topology was identical to those derived from three comprehensive datasets: the complete chloroplast genomes, the 79 plastid CDS, and the 133 IGS regions (Figure 7). This highly stable topology also yielded robust bootstrap support across most internal nodes. Previous foundational studies have successfully clarified the broad generic boundaries and phylogenetic framework of the *Eria* s.l. complex using standard nuclear and plastid markers (Ng et al. 2018). However, such conventional loci frequently encounter limitations in resolving power when differentiating closely related terminal species. Our in silico validation demonstrates that the combination of these seven newly identified loci harbors a significantly denser phylogenetic signal. While future empirical studies involving population‐level PCR amplification will be necessary for ultimate field application (Hollingsworth et al. 2011), this specifically tailored multi‐locus barcode provides a highly sensitive and cost‐effective tool to complement traditional markers for fine‐scale species delimitation within this challenging group.

### 4.5 Adaptive Evolution of Plastid Protein‐Coding Genes

The evolutionary rate and selective pressure acting on CDS provide profound insights into the adaptive mechanisms of plant lineages. In our pairwise Ka/Ks analysis using *P. amica* as a reference, the overwhelming majority of the 79 plastid CDS exhibited Ka/Ks ratios substantially lower than 1 (overall average = 0.2520). This prevailing pattern indicates that strong purifying selection acts to maintain the functional conservatism of the chloroplast genome across the *Eria* s.l. complex, ensuring the stability of the essential photosynthetic apparatus (Weng et al. 2014). Nevertheless, specific genes exhibited notably elevated non‐synonymous substitution rates. For instance, the *ycf1* gene displayed a relatively high evolutionary rate; however, Ka/Ks ratios strictly greater than 1 were only observed in specific pairwise comparisons (e.g., against *C. strictus* and *Trichotosia* species), while remaining below 1 in others (Figure 6, Table S3). While pairwise metrics provide a valuable overview of average selective pressures across entire genes and lineages, evolutionary adaptations can also be driven by episodic positive selection acting on specific amino acid sites within clades (Zhang et al. 2005). To further investigate these fine‐scale evolutionary dynamics, we additionally performed selection pressure analysis using the branch‐site model in PAML. By designating the distinct generic clades as foreground branches, we unequivocally identified a highly significant signal of positive selection (*p* < 0.001) acting exclusively on the *ycf1* gene within the *Pinalia* lineage.

The *ycf1* gene is widely recognized as one of the largest and most rapidly evolving genes within plant plastomes. Accumulating evidence indicates that *ycf1* encodes the TIC214 protein, an indispensable core component of the translocon at the inner chloroplast envelope (TIC) complex. This complex fundamentally regulates the import of numerous nuclear‐encoded precursor proteins that are vital for plastid development, homeostasis, and stress responses (Kikuchi et al. 2013). The detection of strong episodic positive selection on the *ycf1* gene specifically within the *Pinalia* lineage constitutes a compelling evolutionary footprint. Given the specific epiphytic or lithophytic ecological niches occupied by *Pinalia* species, adaptive mutations within the TIC214 translocon might have been favored to optimize the selective import of specific nuclear‐encoded functional proteins under fluctuating environmental stresses, such as intense canopy light or periodic drought (Fan et al. 2018). Consequently, the localized positive selection acting on *ycf1* likely facilitated the successful evolutionary radiation and specific eco‐physiological adaptations of the *Pinalia* clade within the broader *Eria* s.l. complex.

### 4.6 Phylogenetic Relationships and the Rapid Radiation of the *Eria* s.l.

Our phylogenetic analyses strongly supported the monophyly of the *Eria* s.l. complex. Consistent with previous multi‐locus studies (Ng et al. 2018), *Eria* was identified as the basal diverging lineage (estimated at 9.97 Ma). While traditional markers often yielded unresolved, polytomy‐like backbones for the remaining clades (Ng et al. 2018), our plastome‐scale data recovered a highly supported topology for the five sampled genera (Figure 7). Across all evaluated datasets, ML analyses consistently recovered a sister relationship between *Pinalia* and *Trichotosia* (diverging at 5.53 Ma), as well as a clustering of *Cryptochilus* and *Dendrolirium*. Interestingly, the time‐calibrated chronogram inferred a successive divergence for the latter two genera rather than a sister relationship. This topological incongruence is likely attributed to their extremely narrow divergence window (*Cryptochilus* at c. 6.55 Ma, followed by *Dendrolirium* at 6.34 Ma). Such a short interval (c. 0.21 million years) suggests an episode of rapid evolutionary radiation, where lineages lack sufficient time to accumulate synapomorphies, frequently causing soft conflicts between unconstrained algorithms and molecular clock models (Whitfield and Lockhart 2007). While restricted taxon sampling might also partially influence these deep nodes (Zwickl and Hillis 2002), this rapid radiation hypothesis provides a robust evolutionary explanation.

This inferred Late Miocene radiation (9.97–5.53 Ma) coincides with major paleoclimatic events, particularly the intensification of the Asian summer monsoon and the Himalayan–Tibetan plateau uplift (Favre et al. 2015). These profound environmental shifts likely acted as catalysts for rapid diversification, a pattern similarly observed in other Asian epiphytic orchids like *Cymbidium* and *Dendrobium* (Givnish et al. 2015; Xue et al. 2023; Chen et al. 2024). At the infrageneric level, the *Pinalia* lineage underwent recent crown diversification starting around 4.03 Ma, with terminal taxa ( *P. bipunctata* , *P. yunnanensis*, and *P. obvia*) speciating during the Pleistocene (c. 2.51–1.15 Ma). This extremely recent divergence perfectly explains their considerable plastome conservation and low genetic divergence. Overall, our study demonstrates that the generic and infrageneric diversity within *Eria* s.l. was predominantly shaped by climate‐driven rapid radiation.

## 5 Conclusion

In this study, we sequenced and assembled the complete chloroplast genomes of 14 *Eria* s.l. species and performed a comprehensive comparative phylogenomic analysis across 18 representative taxa. Our analysis revealed a highly conserved chloroplast genome macrostructure across these species, characterized by high similarity in GC content, overall gene number, and gene order. We identified significant interspecific variations in genome size and inverted repeat (IR) boundaries, most notably characterized by the lineage‐specific, stepwise degradation of the NDH complex in taxa such as *E*. *corneri* and *E. clausa* . Furthermore, we found strong evidence of episodic positive selection in the *ycf1* gene within the *Pinalia* clade, suggesting its adaptive role in plastid protein import mechanisms within dynamic epiphytic environments. Additionally, our phylogenomic reconstructions and divergence dating provided crucial insights into the backbone relationships of the complex, uncovering an episode of climate‐driven rapid evolutionary radiation during the Late Miocene. Leveraging nucleotide diversity, we identified seven hypervariable regions that hold promise as potential molecular markers for fine‐scale species identification within *Eria* s.l. These findings not only significantly enrich the existing chloroplast genome data for this taxonomically challenging group but also provide a robust foundational framework for understanding its complex evolutionary trajectory.

## Author Contributions

**Meina Wang:** project administration (equal), writing – review and editing (equal). **Xinyi Wu:** data curation (equal), formal analysis (equal), writing – original draft (equal), writing – review and editing (equal). **Jian Li:** project administration (equal). **Tingzhang Li:** data curation (equal), formal analysis (equal). **Fengxia Tang:** resources (equal). **Xiaojuan Duan:** resources (equal). **Wenhui Rao:** resources (equal).

## Funding

This work was supported by the Science, Technology and Innovation Commission of Shenzhen Municipality (Grant No. KCXFZ20211020164200001) and (Grant No. JCYJ20210324123013037).

## Ethics Statement

The plant materials used in the study were collected with permission. The collection of plant materials and use complies with relevant institutional, national, and international guidelines and legislation. This article does not contain any studies with human participants or animals and does not involve any endangered or protected species.

## Consent

The authors have nothing to report.

## Conflicts of Interest

The authors declare no conflicts of interest.

## Supporting information


**Table S1:** Detailed information for the chloroplast genome sequences used in this study.

**Table S2a:** Comparison of the nucleotide variability among genes regions of 17 *Eria* s.l. species.
**Table S2b:** Comparison of the nucleotide variability among IGS regions of 17 *Eria* s.l. species.

**Table S3:** Pairwise non‐synonymous to synonymous substitution rate (Ka/Ks) ratios for 42 shared plastid CDS among *Eria* s.l. species. The Ka/Ks ratios were calculated using KaKs_Calculator under default settings, with *Pinalia amica* designated as the reference sequence.

**Table S4:** Detection of episodic positive selection in 75 chloroplast CDS using the branch‐site model.

**Table S5:** Species and accession information for the 50 plastomes used in phylogenetic analyses, retrieved from NCBI GenBank.

## Data Availability

The raw sequencing datasets generated and analyzed during the current study have been deposited in the Genome Sequence Archive (GSA) at the National Genomics Data Center (NGDC), China, under the BioProject accession number PRJCA059605. All data generated or analyzed are included within the article and the Tables S1–, S5.
